# Mass Screening Strategies for Celiac Disease in Apparently Healthy Children and Adolescents: A Systematic Review

**DOI:** 10.3390/medicina62020246

**Published:** 2026-01-24

**Authors:** Alexandra Mpakosi, Vasileios Cholevas, Andreas G. Tsantes, Argyro Pastrikou, Aikaterini Fragkiadaki, Sofia Zhgabi, Vasiliki Mougiou, Nicoletta Iacovidou, Rozeta Sokou

**Affiliations:** 1 Department of Immunology, General Hospital of Nikaia “Agios Panteleimon”, 18454 Piraeus, Greece; argiropastr@gmail.com (A.P.); kathy_fragi@yahoo.gr (A.F.); 2 School of Medicine, University of Bologna, 40126 Bologna, Italy; billcholevas34@gmail.com; 3 Department of Microbiology, Saint Savvas Oncology Hospital, 11522 Athens, Greece; andreas.tsantes@yahoo.com; 4 Department of Biology, School of Medicine, National and Kapodistrian University of Athens, 11527 Athens, Greece; sofiazhgabi@gmail.com; 5 Neonatal Department, Aretaieio Hospital, National and Kapodistrian University of Athens, 11528 Athens, Greece; vassouli@hotmail.com (V.M.); niciac58@gmail.com (N.I.)

**Keywords:** celiac disease, screening, gluten free diet, rapid test, point of care test

## Abstract

*Background and Objectives*: Celiac disease (CD) is a major global public health problem that can occur at any age. Pediatric CD can be typical, atypical, or even asymptomatic. Early diagnosis and early initiation of treatment are essential for improving patients’ quality of life and preventing serious complications later in life. However, it is impossible to identify asymptomatic children and adolescents without screening. In this systematic review, we attempted to identify different mass screening programs that have been reported for CD in apparently healthy children and adolescents across the world, to highlight the advantages and disadvantages of such strategies, and to collect and synthesize data from these studies reporting the prevalence of CD. In addition, where data were available, we also attempted to evaluate the diagnostic accuracy of the tests used, their cost-effectiveness, the reported clinical benefits, and follow-up data from individuals identified through screening. *Materials and Methods*: Electronic databases, including PubMed and Scopus, were systematically searched. Initially, a total of 316 studies were retrieved. Finally, 55 studies met all inclusion criteria and were included in this review. The included studies were published between 1996 and 2023. *Results*: The reported age of participants ranged from 6 months to 23 years. Confirmation of CD by biopsy was reported in all but six studies. According to the studies that provided data, the (tTG IgA) seroprevalence of CD in apparently healthy children and adolescents, detected through different mass screening methods around the world, ranged from 0.20% (Turkey) to 3.11% (Italy). In addition, the prevalence of biopsy-confirmed CD ranged from 0.036% (Vietnam) to 3% (Sweden and Spain). Studies from 17 countries reported mass screening strategies based on finger-prick rapid tests. All rapid tests detected CD antibodies, except two, which detected HLA DQ2/DQ8 haplotypes. Rapid tests appeared to be no less sensitive and specific than other screening tests for CD and were probably less expensive, but further studies are needed for more reliable conclusions. Of the 55 studies in the review, only 10 reported follow-up data. After 3 months of a gluten-free diet, the general condition of the patients improved; after 6 months, tTG IgA and EMA IgA levels decreased and hemoglobin values increased; while after 1 year, tTG IgG levels also decreased, symptoms subsided, the children’s weight and height increased, school performance improved, episodes of upper respiratory tract infections decreased, and thyreoperoxidase antibodies that were positive at screening became negative. *Conclusions*: Mass screening for CD in asymptomatic children and adolescents is a challenge. Future research should provide more answers regarding the most appropriate target age, the frequency of screening, the optimal screening method, the cost-effectiveness, the clinical utility, and the long-term impact of mass screening on patients’ quality of life.

## 1. Introduction

Celiac disease (CD) is a chronic inflammatory gastrointestinal disease caused by exposure to gluten in genetically susceptible individuals carrying human leukocyte antigen (HLA)-DQ2 or DQ8. It is considered a major public health problem worldwide. The global prevalence of the disease has been found to be 1% [1]. Celiac disease can occur at any age. In very young children, it usually begins within the first 2 years of life, when gluten is first introduced into their diet. The manifestations of pediatric CD can be typical gastrointestinal, such as diarrhea, abdominal distension, and abdominal pain, in both younger and older children, as well as atypical extraintestinal, such as irritability, weight loss, short stature, delayed puberty, anemia, recurrent aphthous stomatitis, high blood transaminases, osteopenia, dental enamel defect, epilepsy, ataxia, headache, depression, arthritis, and dermatitis herpetiformis, mainly in older children and in cases diagnosed late. In addition, many cases are asymptomatic [2,3]. In fact, CD has been likened to the “iceberg model,” with symptomatic patients constituting only 10% of the iceberg and the remaining 90% consisting of patients with an atypical and silent form of the disease [4].

After positive serology for CD, an intestinal biopsy is required to confirm the diagnosis. However, the European Society of Paediatric Gastroenterology, Hepatology and Nutrition (ESPGHAN) set guidelines, first in 2012 and then revised in 2020, regarding a biopsy-free approach for children who meet specific criteria. Thus, according to the updated 2020 ESPGHAN guidelines, the criteria should be an IgA antibodies to tissue transglutaminase (tTG IgA) level ≥ 10 times the upper limit of normal (ULN) and positive endomysial IgA antibodies (EMA IgA) for all children, both symptomatic and asymptomatic). In contrast to the old 2012 guidelines, the criterion for HLA typing was omitted [5,6].

A lifelong gluten-free diet (GFD) usually improves most symptoms and provides relief to patients. Moreover, according to current recommendations, high-risk groups for developing the disease should be screened. These include first-degree relatives, people with selective IgA deficiency, type 1 diabetes, autoimmune thyroid disease, gastric autoimmunity leading to pernicious anemia, vitiligo, adrenal insufficiency, Down syndrome, Turner syndrome, and William–Beuren syndrome [7]. However, there are still no clear recommendations regarding screening for asymptomatic (silent) patients with CD [8]. Previous screening studies have shown that 50% to 70% of patients diagnosed with CD were asymptomatic [9]. In addition, a large percentage of children, at least 91% from socioeconomically disadvantaged backgrounds and at least 83% from socioeconomically privileged backgrounds, remain undiagnosed [10]. This may be due in part to the absence of clear manifestations of the disease. However, it is impossible to identify asymptomatic children without screening. Screening leads to early diagnosis and early initiation of a GFD, improving the quality of life of patients even if patients are asymptomatic. Conversely, prolonged delay in diagnosis is associated with an increased risk of complications such as infertility, osteoporosis, and reduced quality of life [11]. An increased risk of serious complications, such as the development of cancer, has also been observed in cases that remain undiagnosed for a long time [12]. Furthermore, it has been reported that the mortality risk of undiagnosed CD patients may be considerably higher than that of the healthy population [13]. From a community perspective, it appears that delayed diagnosis of CD is associated with increased medical costs. Moreover, gluten-free products are more expensive than products containing gluten. This additional cost is charged to the patient in some countries, while in others it may be partially covered by the health system or insurance [14]. Therefore, undetected CD is burdensome from both patient and community perspectives and intensifies the need for early diagnosis.

Celiac disease appears to meet the most important criteria defined by the World Health Organization (Wilson and Jungner criteria) for screening. It is a disease that constitutes a significant health problem, there is a recognizable preclinical stage, there is an appropriate and acceptable test, and there is an acceptable treatment for patients [14]. Several studies from different countries have shown that implementing mass screening for CD can lead to early diagnosis of asymptomatic children, who, after following a GFD, will be able to avoid possible future complications. Italy is the only country that decided in 2023 by law to implement nationwide mass screening for CD and type 1 diabetes (T1D) in all children aged 1–17 years [15]. On the other hand, other studies present opposing views. Thus, such strategies provoke debate and remain controversial. In this systematic review, different mass screening programs reported for CD in apparently healthy children and adolescents are identified and their outcomes are examined. The advantages and disadvantages of such strategies are also highlighted. The primary objective of this systematic review was to collect and synthesize evidence from studies reporting the rates of CD diagnosis in the general pediatric population following the implementation of mass screening programs. The secondary objectives comprise, where evidence is available, the evaluation of the diagnostic performance of the serological assays utilized, the characterization of the applied screening protocols, the delineation of diagnostic confirmation criteria, and the synthesis of reported clinical benefits, potential adverse outcomes, and longitudinal follow-up data of children identified through screening.

## 2. Materials and Methods

A systematic review methodology was undertaken to comprehensively identify, appraise, and synthesize studies addressing the predefined research objectives. EndNote X8 Clarivate Analytics (US) software was used. The review protocol was developed in strict adherence to the Preferred Reporting Items for Systematic Reviews and Meta-Analyses (PRISMA) guidelines (Table S1; presented as a Supplementary Material) and was prospectively registered with the International Prospective Register of Systematic Reviews (PROSPERO CRD420251167715) [16]. 

### 2.1. Information Sources and Search Strategy

A systematic review of the literature was conducted during August 2025, with a search end date of 19 October 2025. Electronic databases, including PubMed and Scopus, were systematically searched. The search strategy used the Boolean operators OR and AND and included the following keywords and phrases: “children,” “school,” “mass screening,” “pediatric celiac disease,” “pediatric coeliac disease,” “celiac schoolchildren,” “coeliac schoolchildren,” “celiac disease,” “coeliac disease,” and “child.” Additionally, to minimize the risk of missing relevant studies and to comprehensively cover the entire scope of the available literature, the bibliographic references of each included study were searched and reviewed, as well as references from previous systematic reviews in the same research field.

### 2.2. Inclusion Criteria

All studies included in our systematic review were required to meet the following predefined inclusion criteria, which were structured according to the population, intervention/exposure, outcomes, and study design.

#### 2.2.1. Population

- The review included studies involving children and adolescents (school-aged or within the broader pediatric spectrum as defined by each study), asymptomatic or without a prior diagnosis of celiac disease, recruited from the general population (e.g., schools and community-based cohorts).

#### 2.2.2. Intervention/Exposure

- Studies examined mass or organized screening programs for celiac disease in unselected pediatric populations, including school-based and population-level initiatives.
- Eligible screening modalities comprised serological testing (anti-TGA IgA, EMA IgA, and total IgA), blood-based or point-of-care assays, as well as combinations of these approaches.

#### 2.2.3. Comparators

- A comparator group was not required for inclusion. When studies provided comparative data, such as screening versus no screening or comparisons of different test modalities, these data were extracted and analyzed.

#### 2.2.4. Outcomes

We included all studies that provided data on our primary outcome. Secondary outcomes were also reviewed and extracted where such information was available in the original reports.

*Primary outcomes*

- Detection rate or prevalence of celiac disease identified through mass screening including serology-positive cases confirmed via biopsy or according to ESPGHAN diagnostic criteria-confirmed cases).

*Secondary outcomes*

- Diagnostic accuracy measures of the employed tests (sensitivity, specificity, positive predictive value, and negative predictive value) against the reference standard (intestinal biopsy or ESPGHAN 2020 biopsy-free criteria).
- Rate of diagnostic confirmation (percentage of screen-positive cases proceeding to biopsy or ESPGHAN-based diagnosis).
- Proportion of children initiating a GFD after diagnosis.
- Health-related outcomes post-diagnosis (e.g., symptom resolution, growth parameters, hematological indices, and bone mineral density), where available.
- Reported harms or adverse consequences (e.g., psychosocial burden, necessary versus unnecessary biopsies, economic costs, and false-positive and false-negative classifications).
- Follow-up adherence and attrition rates.

#### 2.2.5. Study Design

- Included study designs encompassed observational studies (cross-sectional and cohort), mass screening programs with reported outcomes, pilot or population-based initiatives, and epidemiological surveys incorporating screening data.

### 2.3. Exclusion Criteria

The following exclusion criteria were applied to ensure that studies that were not aligned with the scope or methodological requirements of this review were omitted:

- Studies conducted exclusively in first-degree relatives, individuals with established autoimmune disorders (such as type 1 diabetes or autoimmune thyroid disease), genetic syndromes (e.g., Down or Turner syndrome), or other high-risk cohorts undergoing targeted screening were excluded.
- Case reports, small case series without defined screening methodology, and studies limited to targeted or high-risk populations, as well as reviews or meta-analyses.
- Studies published only as conference abstracts or in languages other than English were excluded, while no geographical or temporal restrictions were applied.

### 2.4. Data Extraction and Resolution of Disagreements

Screening, data extraction, and quality assessment were conducted independently by two researchers (A.M. and A.G.T.). Any conflicts were resolved through discussion and consensus between the researchers or, when necessary, by a third researcher (R.S.).

Data from the selected studies were extracted and recorded in a tabular format, including the following fields: first author, study design, year of publication, country of study, title, authors, implementation level (school or national), age range and number of participants, method of invitation and participation, screening type and protocol (test and cut-off), total number of positive serology results, number of confirmed cases (biopsy or ESPGHAN criteria), positivity rates, follow-up data, reported harms or psychosocial outcomes, costs (where reported), and methodological details (study design, study period, and response rates). Additionally, for diagnostic criteria, it was recorded whether the diagnosis was based on biopsy or followed the ESPGHAN 2020 biopsy-free criteria.

## 3. Results

**Figure 1:**
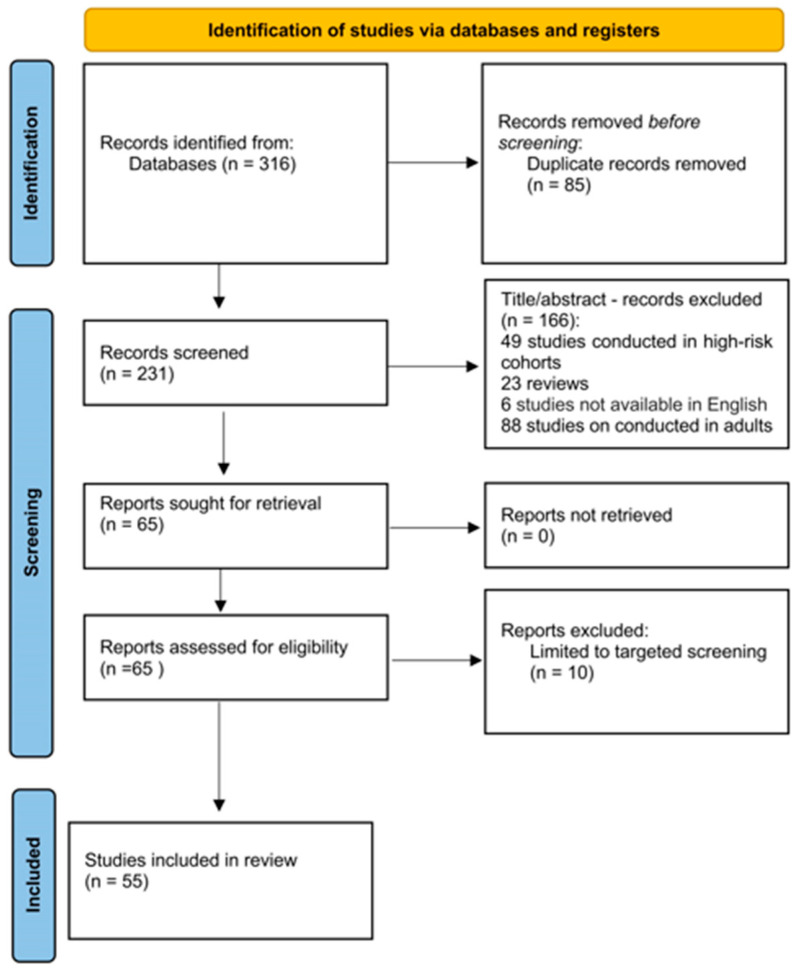
Flow diagram of the study selection process.

A total of 316 studies were retrieved from the search in electronic bibliographic databases. Of this total, 85 were duplicates and were removed. The removal of duplicates was accomplished with the help of the duplicate work removal tool in the bibliographic citation program (EndNote X8). After carefully reading the titles and abstracts of the remaining 231 studies, 166 studies were excluded either because their subject matter did not serve the purpose of the study or they met some of the exclusion criteria in the title and abstract. After carefully reading the full text of the 65 remaining studies, 55 studies met all inclusion criteria and were included in this review [9,17,18,19,20,21,22,23,24,25,26,27,28,29,30,31,32,33,34,35,36,37,38,39,40,41,42,43,44,45,46,47,48,49,50,51,52,53,54,55,56,57,58,59,60,61,62,63,64,65,66,67,68,69,70] (Table 1). The flow chart is presented in Figure 1.

The included studies were published between 1996 and 2023. Studies were conducted in Italy (*n* = 9), Turkey (*n* = 6), Sweden (*n* = 4), Saudi Arabia (*n* = 3), India (*n* = 3), Spain (*n* = 2), the United States (US) (*n* = 2), Iran (*n* = 2), Finland (*n* = 2), Tunisia (*n* = 2), and the Netherlands (*n* = 2), with single studies from Croatia, San Marino, Russia, Jordan, Estonia, Brazil, the northern region of Cyprus, Belgium, the United Kingdom, Greece, Germany, Vietnam, Egypt, Hungary, Argentina, Malta, Slovenia, Switzerland, Colombia, Libya, and Hungary. The reported age of participants ranged from 6 months to 23 years (Table 1).

**Figure 2:**
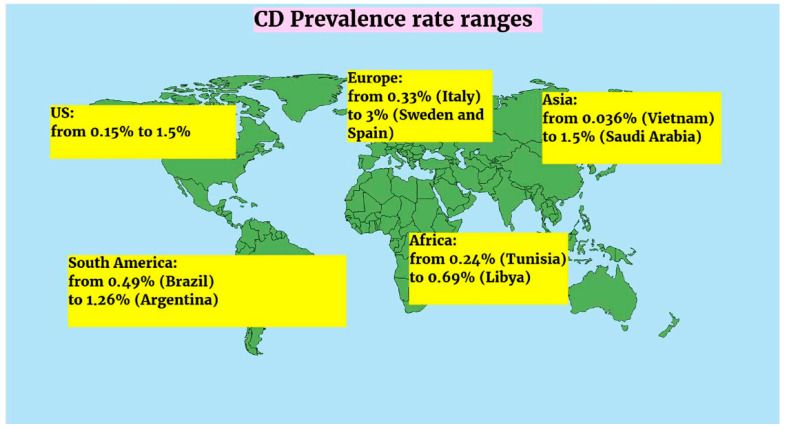
Global ranges of CD prevalence rates (according to the studies included in the review). CD: celiac disease.

The observed prevalence rates indicated significant inter-regional heterogeneity. Europe consistently reported the highest burden of celiac disease. In the present study, the prevalence rates ranged from 0.33% (Italy) to 3% (Sweden and Spain), whereas in the United States it ranged from 0.15% to 1.5%, in South America it ranged from 0.49% (Brazil) to 1.26% (Argentina), in Asia it ranged from 0.036% (Vietnam) to 1.5% (Saudi Arabia), while in Africa it ranged from 0.24% (Tunisia) to 0.69% (Libya), highlighting substantial global variation (Figure 2).

**Figure 3:**
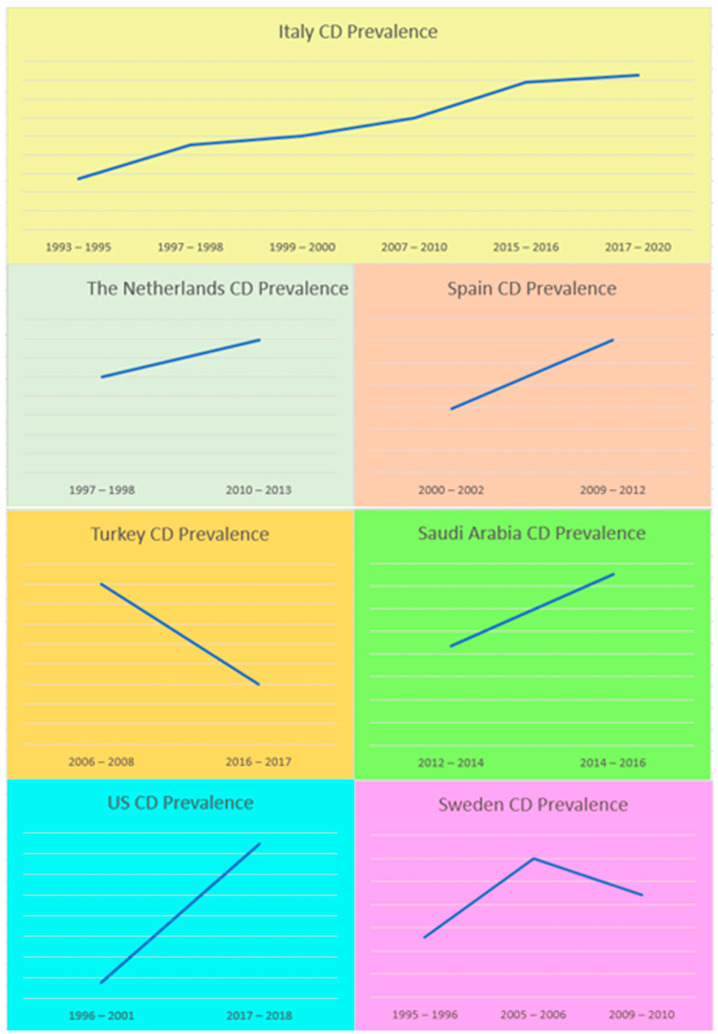
CD prevalence trend by country (according to the studies included in the review). CD: celiac disease, US: United States.

For countries where two or more studies were available, graphs were created. Among these, Italy showed a continuous increase in the prevalence of CD in children and adolescents in recent years (Figure 3).

The diagnostic accuracy of the tests used is shown in Table 2. The four rapid tests (Biocard, Biohit, Operon, and salivary test) appeared to be no less sensitive and specific than the other screening tests for CD. However, this information was obtained from studies that provided such data. Therefore, more studies are needed for confirmation.

In addition to being sensitive and specific, the screening test for CD must also be inexpensive (Table 3).

Studies from 17 countries reported mass screening strategies based on finger-prick rapid tests. All rapid tests detected CD antibodies, except two tests that detected HLA DQ2/DQ8 haplotypes (Table 4).

Of the 55 studies included in the review, only 10 reported follow-up data (Table S2; presented in Supplementary Materials).

## 4. Discussion

**Figure 4:**
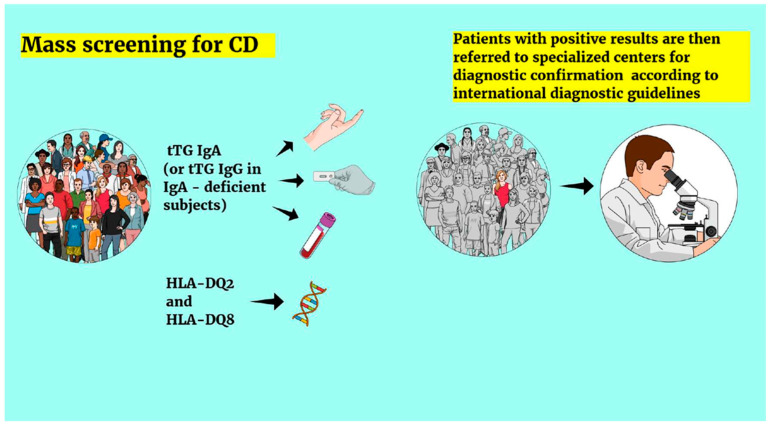
Main screening pathways for CD (as described in the studies included in this review). CD: celiac disease, tTG: tissue transglutaminase, HLA: human leukocyte antigen.

This study provides a comprehensive overview of mass screening strategies for celiac disease in apparently healthy children and adolescents worldwide. The main screening pathways, as described in the studies included in this review, are summarized in Figure 4.

By synthesizing data from diverse populations, screening methodologies, and healthcare settings, the study demonstrates substantial variation in seroprevalence, biopsy-confirmed disease rates, and follow-up outcomes. These findings highlight both the potential benefits and the challenges of population-based screening and offer important insights into the epidemiological, clinical, economic, and ethical implications of early detection of CD in the pediatric population. Interpreting these findings therefore requires careful consideration of both biological and methodological factors when assessing the potential role of mass screening strategies in pediatric populations.

According to the studies that provided data, the (tTG IgA) seroprevalence of CD in apparently healthy children and adolescents, detected through different mass screening methods around the world, ranged from 0.20% (Turkey) to 3.11% (Italy). In addition, the prevalence of biopsy-confirmed CD ranged from 0.036% (Vietnam) to 3% (Sweden and Spain). The observed rate ranges indicate significant global variation. Biopsies were not performed in six studies [20,21,52,53,69,70]. The higher prevalence rates of CD in developed countries compared to developing ones has been previously reported [71]. This difference may be due to many factors, including genetic and environmental factors, such as dietary behaviors, differences in gluten consumption, age of gluten introduction, neonatal and childhood infections, antibiotics and proton pump inhibitor (PPI) medications, infant feeding practices, and gut microbiome synthesis [72]. Furthermore, in recent years, the consumption of food products containing additives has increased, especially in developed countries. It is noteworthy that between 2001 and 2019 in the US, the percentage of baby foods purchased containing additives increased by 20% and the percentage of purchases containing three or more additives increased by >15% [73]. Among them, microbial transglutaminase (mTG) is a widely used additive in the food industry. The enzyme belongs to the transglutaminase family and mimics the post-translational modification of gluten by tissue transglutaminase, possibly playing an important role in the induction of CD [74]. However, this difference in prevalence rates may be false. It is possible that in developed countries more screening programs have been organized, while residents are more informed about the disease and more available for diagnostic tests. The majority of the studies in this review came from the developed world. On the other hand, in developing countries the disease is likely to be underdiagnosed, due to both insufficient awareness among the population and healthcare personnel and the lack of resources. For example, data from the Sub-Saharan region have not been reported. Therefore, Africa is represented almost exclusively by North African countries. However, the populations of North African countries are genetically, culturally, nutritionally, and epidemiologically different from the populations of Sub-Saharan Africa. Therefore, it can be assumed that generalizing these findings to the whole of Africa may be misleading. Nevertheless, it has been previously suggested that the population prevalence of the HLA-DQ2 genotype and gluten consumption are lower in Sub-Saharan Africa than in North Africa; therefore, the prevalence of CD is likely to be lower as well [71]. Furthermore, this review presents data from only 4 of the 10 most populous countries in the world (India, the United States, Brazil, and Russia), while no data were available from the remaining six countries (China, Indonesia, Pakistan, Nigeria, Bangladesh, and Japan), as in the review by Singh et al., who conducted a systematic review and meta-analysis to estimate the global prevalence of celiac disease [71].

When data were available from more than one study from the same country, these data were used to examine the trend in prevalence over time, and an upward trend was observed, which should however be treated with caution. This is because comparing the prevalence of CD from different cities, groups, or age groups at different points in time does not allow for drawing firm conclusions about time trends and the national prevalence of CD. Such conclusions require repeated measurements in the same or comparable populations using identical methodologies. Furthermore, the upward trend observed in most of these graphs may be related, on the one hand, to improved and/or wider availability of diagnostic methods and, on the other hand, to greater awareness and/or availability of the screening population. In Italy, however, the upward trend could reflect a real increase in the prevalence of CD in children and adolescents over the last 27 years, as Gatti S. et al. have also argued (available data from nine different studies published between 1996 and 2023) [19]. According to Gatti S. et al., genetic, dietary, and environmental factors could possibly be involved [19]. As previously mentioned, the prevalence of CD may also vary even between regions of the same country, possibly due to differences in gluten consumption, such as between central and southern Italy [18,21]. It is worth noting that most of the studies in this review came from Italy, which is the only country that approved by law in 2023 the implementation of screening for CD and T1D in children aged 1–17 years [15,75]. The nationwide screening program will begin in 2026, following a pilot study (the D1Ce Screen) in four regions, with the participation of 5363 children aged 2, 6, and 10 years who were tested for HLA genotypes and autoantibodies for CD and T1D [76]. Six studies were from Turkey, but only two had data available for the study period. Therefore, the graph most likely does not reflect a true downward trend in CD prevalence [31,32]. In the graph for Sweden, an upward trend in the prevalence of CD was recorded, reaching up to 3% in the period 2005–2006, which may be due to the feeding practice of the abrupt introduction of gluten, without continuous breastfeeding in infants, followed by a downward trend (prevalence of CD 2.2%) in the period 2009–2010 when this practice changed [66,68].

Of the 55 studies in the review, only 10 reported follow-up data [18,19,27,29,35,38,43,49,50,54]. Four of these studies came from Italy [18,19,35,50], and the rest came from Finland [27], Switzerland [29], Hungary [38], India [43], the Netherlands [49], and the USA [54]. In some studies [18,19,27,54], children who tested positively in the initial screening test were subsequently negative during the follow-up and while on a gluten-containing diet (transient tTG IgA positivity). In addition, children who tested negatively for tTG IgA at the initial screening developed CD within 3 years of their negative screening result at age 6 [49]. These results should be taken into account when discussing the optimal age and frequency of screening for CD. On the one hand, early screening is preferable to prevent serious complications later, but on the other hand, early screening with a negative initial test could miss some patients [31]. For example, the age of 2–3 years has been suggested as the most appropriate time to screen for CD by measuring tTG antibodies. Indeed, tTG IgA seroconversion tends to peak at the age of 2 to 3 years and plateaus by age 10. However, it should not be ignored that the disease can manifest at any age, and seroconversion may happen at any age as well; therefore, a single negative result should not lead to complacency [64].

After 3 months on a GFD, an improvement in the general condition of the patients was reported [29]. After 6 months on a GFD, tTG IgA and EMA IgA levels decreased significantly and hemoglobin values increased significantly [35,38]. tTG IgG levels decreased within the first 12 months [50]. After 1 year on a GFD, symptoms resolved, children’s weight and height increased, school performance improved, episodes of upper respiratory tract infections decreased, and thyreoperoxidase antibodies that were positive at screening became negative [35,50]. No complications due to the disease were observed in children following a GFD [35]. A gluten-restricted diet was also able to induce clinical improvements in patients [43]. On the other hand, persistent low tTG IgA positivity after 18 months of a GFD was likely due to suboptimal compliance of the children [50]. Despite their benefits, gluten-free products are expensive and may not be available to everyone. As a result, some children who tested positive and started a GFD had difficulty maintaining it [29]. Furthermore, it is challenging to convince asymptomatic children to follow a GFD and return for follow-up testing [54]. In any case, more studies need to be conducted on the long-term follow-up of patients in order to draw more precise conclusions regarding the impact of screening on children’s quality of life.

Mass screening programs seek to identify people who, although often not showing symptoms, already have a disease. They offer the possibility of referring those who test positive via screening for further tests aimed at early diagnosis and treatment in order to reduce the risk of complications of the disease. As previously mentioned, according to the criteria established by the World Health Organization (WHO) regarding screening programs, the disease should be a major health problem and the screening, diagnosis, and treatment procedures should be well understood and accepted by the participating population [77]. Indeed, when apparently healthy individuals are involved, certain ethical issues may arise. For this reason, comprehensive information exchange from those responsible for screening to participants is imperative, including all of the parameters of the procedure, as well as the risks of a possible late diagnosis on the one hand, and the benefits of accelerating treatment on the other. The challenge is even greater when screening programs target children and adolescents who do not have the ability or the right to decide on their participation [78]. Alessandrini S. et al. showed that the screening program itself was a cause of public awareness about CD. Thus, restaurants began to include gluten-free menus while schools established mandatory special lunches for children with CD [17].

This review demonstrates that, although many seropositive children and adolescents declared themselves to be asymptomatic, they actually had delayed physical development and had more frequent iron deficiency anemia, malnutrition, osteopenia, and low folate concentrations than seronegative children of the same age [9,33,45]. However, other studies in the review emphasized that low height and weight do not always indicate CD and that individuals with normal or even excess weight are at the same risk of developing the disease [9,22,29]. Furthermore, Rutz R. et al. found no difference in height, weight, stool habits, and episodes of abdominal pain between students with elevated and normal CD autoantibodies [29].

Mass screening programs are expensive and difficult to organize. Therefore, to implement mass screening, it is necessary to balance the benefits of early diagnosis (e.g., avoiding long-term complications) with the costs. Several studies showed that mass screening for CD was associated with improved quality adjusted life-years (QALYs) and was a cost-effectiveness strategy. Hershcovici T. et al. used a Markov model to show that the incremental cost-effectiveness ratio (ICER) of the screening strategy versus the no-screening strategy was 48,960 USD/QALYs [79]. The variables that most affected cost-effectiveness were the time delay from symptom onset to diagnosis, the adherence to a GFD, and the prevalence of CD. They also argued that screening would be cost-effective if the time delay to diagnosis is greater than 6 years, given that such a long delay can lead to increased morbidity and mortality [79]. Furthermore, Heijdra Suasnabar J. et al. estimated the long-term cost-effectiveness of case finding and mass screening strategies for CD in children, also considering their impact on long-term costs and QALYs. They showed that both strategies were highly cost-effective compared to clinical diagnosis and concluded that, between them, mass screening is likely the optimal strategy [80]. Long K.H. et al. evaluated the impact of a CD diagnosis on healthcare costs and additional costs associated with the disease and showed that the average total cost was reduced by $1764 in the year after diagnosis [81]. Greco L. et al. showed that the estimated standardized medical costs for children with symptomatic celiac disease during the delay between symptom onset and diagnosis (mean of 2 years) will be approximately 387 million euros over the next 10 years [82]. They also argued that a delay in diagnosis is expected to increase mortality, and that approximately 600,000 patients with CD will die in the next 10 years, representing a more than 44.4% increase compared to individuals of similar age and sex [82]. A recent review revealed that most of the economic burden is due to outpatient care. Therefore, early initiation of a GFD may lead to a reduction in visits to primary care providers and missed days of school and work [83]. However, in the US, insurance companies refused to pay the cost of biopsies for 21% of patients who were seropositive at screening on the grounds that they had no symptoms, leading to a potential risk of delayed diagnosis [41]. Another recent study estimated the potential costs associated with delayed diagnosis of the disease in Iran. It showed that while the average age of onset of the first symptoms of the disease was 12 years, diagnosis was not made before the average age of 22 years. The estimated total cost was mainly due to the delayed diagnosis of CD and possible complications and included diagnostic tests, hospitalization costs, medical visits, travel costs, medications, and lost productivity (on average 10 lost workdays) [84]. This may be more economically burdensome in low-income countries where there is a longer delay in the diagnosis of CD (on average 10 years, as previously mentioned) compared to 5–7 years in high-income countries [84]. The standard of living, nutrition, medical care, and education of these residents lag behind those of high-income countries, contributing to the lack of information and ignorance of the population regarding the mosaic of symptoms of CD [77]. But even within the same country, people with higher education and socioeconomic status tend to be more proactive in seeking diagnostic tests and adapt more easily to undergoing follow-up tests and following a GFD [84]. On the contrary, Kondrashova A et al. argued that worse living conditions and hygiene may act protectively against the disease [63].

As can be seen from the above discussion, determining the cost-effectiveness of a screening program is challenging because improvements in health-related quality of life, as measured by QALYs, must be compared with costs and savings [85]. Indeed, comprehensive health economic evaluations of CD screening are very limited. However, as previously mentioned, mass screening for CD is likely to be cost-effective. Shamir R. et al. showed that screening was cost-effective in populations of a wide age range with a relatively high prevalence of CD or when the SMR (standardized mortality ratio) for untreated CD patients was high [86]. However, they reported that the Markov model they developed was not sensitive to changes in the costs of serological markers and diagnostic endoscopy [86]. Park K.T. et al. evaluated the cost-effectiveness of universal serologic screening (USS) versus symptomatic at-risk screening (SAS) strategies for CD due to the risk of non-traumatic hip and vertebral fractures in cases of untreated or undiagnosed cases [87]. The authors developed a Markov model for lifetime screening strategies, each with groups of 1000 patients, of both sexes, aged 12 years when screening began. They showed that the SAS strategy was overall more cost-effective in preventing bone loss and fractures in patients with undiagnosed or subclinical disease [87]. Norström F. et al. showed that mass screening for CD may be cost-effective under various circumstances, especially with regard to the natural history of celiac disease (for example, rapid progression from asymptomatic to symptomatic CD or long delay from symptomatic CD to diagnosis) and the long-term health complications of CD patients who still follow a gluten-containing diet [88]. In a more recent study, a cost analysis was conducted at a regional level, over a ten-year time horizon with a target population of children aged 2, 6, and 10 years who underwent serological testing and HLA genotyping. The study concluded that while screening dominates the initial cost, the cost of a GFD constitutes the main financial burden throughout life [89]. Despite the positive findings, there is still no consensus or guideline for mass screening for CD in the general population. Therefore, there is a need for further studies evaluating the clinical utility, cost-effectiveness, and long-term outcomes of screening strategies in asymptomatic individuals.

Mass screening for CD in children has multifaceted effects on the psychological well-being of both children and their parents. Data indicate that diagnosis via screening often initially elicits surprise and anxiety, but most parents and children express gratitude for early identification of the disease, as it leads to improved quality of life following the initiation of a GFD [78,90].

Specifically, the psychological burden is greatest at the time of diagnosis, with elevated levels of anxiety and depressive symptoms observed in both children and parents compared to healthy populations. However, after one year of treatment, significant improvements in symptoms and quality of life are noted, without an increase in psychological problems. Informing parents about the diagnosis appears to reduce reported psychological symptoms in children, suggesting that awareness and support play a crucial role [90,91,92,93].

Compliance with the diet is generally high and screening is positively received by the majority of families, although some express ambivalence regarding the personal benefits and lifelong challenges of dietary adherence. Long-term follow-up indicates that the health and psychological status of children diagnosed via screening align closely with those of the general population [78,94].

In summary, mass screening for CD may initially induce psychological stress, but it ultimately leads to improvements in quality of life and psychological well-being for both children and parents, with high acceptance and adherence to the dietary regimen [78,90,91,92].

The screening test should be inexpensive, simple, valid, reliable, and with minimal inconvenience to the population. For example, capillary blood may be preferable to venous blood sampling, which is less acceptable, especially by children, and more time-consuming and expensive, as it involves transportation and analysis in the laboratory. Studies from 17 countries included in this systematic review reported mass screening strategies based on finger-prick rapid tests. The majority were from Italy (*n* = 4) and Turkey (*n* = 4). The rest were from Malta, Greece, Libya, Tunisia, Croatia, Spain, Hungary, Slovenia, and Colombia. All screening tests detected celiac antibodies except for two of the four Italian studies, which detected HLA DQ2/DQ8 haplotypes [18,19]. These finger-prick rapid tests are easy to perform, even by non-medical personnel, in non-hospital settings that are familiar to children, such as the school environment. However, since these are qualitative tests, false-positive and false-negative results cannot be ruled out [48]. For example, there have been cases in which the nurses who conducted the test may have misinterpreted faint lines of the test as negative results, falsely leading to low sensitivity [38]. By contrast, in Slovenia, supervision of the rapid test procedure by an experienced pediatric gastroenterologist led to more accurate interpretation of faint test lines [40]. Particularly in low-income countries, supervision of the testing process by trained personnel could help avoid the unnecessary costs of false-positive results [40]. Furthermore, two Italian studies suggested the saliva test as a simple, easily accepted by children, and sensitive screening test for CD [35,47].

In general, rapid tests are much cheaper than other assays for diagnosing CD and therefore may be ideal for mass screening programs in countries with limited resources [17,34,38]. To their advantages, one can add the benefits resulting from an immediate response that would potentially reduce the time to biopsy, with subsequent faster diagnosis, treatment, and improved quality of life. Furthermore, when the procedure is performed in a school environment, no school days are lost for students nor workdays for parents (indirect costs) [84]. Several studies have reported high sensitivity, specificity, and positive and negative predictive values for rapid tests compared to other CD tests (tTG and EMA). In addition, the rapid test that detected HLA-DQ2 and DQ8 showed a very high negative predictive value (close to 100%). By contrast, the low positive predictive value was due to the high proportion of HLA-DQ2/DQ8-positive individuals in the general population (Table 5) [18,19].

Strategies using combinations of serological tests (tTG and/or EMA) plus HLA may be more cost-effective than using serological tests alone [95]. Such combinations were reported in this review [18,19,27,34,53,63]. However, mass screening strategies based on HLA-DQ testing have raised some ethical and economic issues. The cost of genetic testing is quite high. In addition, a positive result could psychologically burden healthy HLA DQ2/DQ8 individuals, although such positivity is only associated with a twofold increase in the risk of CD [19]. HLA screening in neonates has also been proposed, although such a strategy is controversial and raises major ethical concerns [29].

As highlighted in this review, tTG antibodies remain the gold standard for CD screening, although it should be noted that no meta-analysis was conducted. However, the combination of tTG IgA plus EMA is superior for diagnosis compared to tTG IgA alone [32]. In cases presenting isolated tTG IgA positivity (with EMA negativity), the ESPGHAN guidelines recommend monitoring patients with a gluten-containing diet and retesting tTG IgA every 3–6 months [18,19].

As previously mentioned, biopsies were not performed in six studies. The no-biopsy approach could potentially reduce diagnosis time, be more acceptable to patients and their parents, increase patient satisfaction, and reduce healthcare costs [96]. Nevertheless, the diagnosis of CD based on serology alone should be made by a pediatric gastroenterologist who should adequately inform the family about the benefits and potential risks of each method [72]. As shown in this review, there were differences between the seroprevalence of CD and the prevalence of biopsy-confirmed CD, most likely due to false-positive screening tests. In some studies [18,19,27,54], children who were positive in the initial screening test were subsequently negative during the follow-up and while on a normal diet (transient tTG IgA positivity). Treatment of potential CD (PCD), which is defined by positive serum antibodies and HLA-DQ2/DQ8 haplotypes but with mild signs of inflammation of the small intestinal mucosa (Marsh score 0–1), poses another challenge for the no-biopsy approach. In this case, it was reported that the presence of symptoms was the main determining factor for starting a GFD [97]. In addition to diagnostic accuracy, the benefits of biopsy could also include the possible diagnosis of coexisting diseases and monitoring the long-term compliance of patients with a GFD [72].

## 5. Limitations of the Study

This systematic review has several important limitations that should be acknowledged. First, due to substantial heterogeneity among the included studies—including differences in sample size, target age groups, screening tests, study design, geographic representation, and duration of the screening programs—a quantitative meta-analysis was not feasible. Therefore, the review presents only a qualitative synthesis of the available data, focusing on the rates of celiac disease diagnosis and characteristics of screening programs rather than quantitative effect estimates. Variations in target age groups and in methods for reporting outcomes further complicated comparisons across studies.

Second, differences in study design and reporting methods, as well as the lack of standardized instruments and follow-up intervals, may have introduced inconsistencies in reporting diagnostic outcomes and follow-up data.

Third, geographical representation was limited, with most studies conducted in specific countries (e.g., Sweden, the USA, and the Netherlands), which may not reflect differences in healthcare systems or screening practices globally.

Fourth, only studies published in English were included, which may introduce a language bias and exclude relevant data from non-English-speaking countries.

Fifth, there was insufficient information on the screening costs. Furthermore, screening costs can vary significantly by country, healthcare system, procurement contracts, and manufacturer. Therefore, the screening costs should be thoroughly evaluated in future studies, which should take into account medical costs, logistics, confirmatory tests, follow-up, and false-positive consequences. Modeling of cost per case detected, cost per QALY, and budget impact should also be thoroughly evaluated.

Sixth, the review included both mass screenings in schools and screenings in the general pediatric population. However, we were careful to include hospital-based studies in which participants did not belong to risk groups nor had symptoms and a previous diagnosis of CD. For example, the participants in these studies were children who underwent blood draws for any reason (mainly in outpatient blood drawing laboratories) or who had undergone blood draws for preoperative tests and for physical fitness certificates for sports. Given that there is limited availability of data in the international literature regarding the prevalence of CD in apparently healthy children and adolescents, we believe that each of such studies adds important information and raises awareness.

Seventh, the review covered almost three decades (1996–2023), during which diagnostic tests, thresholds, awareness, and guidelines have changed significantly. Changes in methodology over time likely explain some of the observed variations. However, there is a lack of sufficient data, especially from older studies.

Eighth, screening studies with low participation may have underestimated or overestimated the true prevalence, yet response rates were not adequately reported in the studies included in the review.

Ninth, further studies are needed on the diagnostic accuracy of different screening tests. The analytical performance of the tests themselves must also be taken into account. For example, IgA anti-TG2 assays have shown significant variation over the years. Even when using the same platform, results can differ between kits from different manufacturers, and there may also be variation in the cut-off values used to define positivity in screening algorithms. This inherent variability may also contribute to differences in reported screening outcomes. In addition, it needs to be more reliably confirmed whether rapid tests are as sensitive and specific as standard assays.

Tenth, because follow-up data were reported for only a minority of studies, conclusions regarding clinical benefit and improvement in quality of life should be treated with caution. Further studies are needed to obtain more reliable conclusions.

Finally, the absence of randomized controlled trials and quantitative meta-analysis precluded statistical synthesis of diagnostic rates or screening performance metrics.

Despite these limitations, this review provides a comprehensive overview of available evidence on mass screening for CD in children, including diagnostic rates, screening protocols, and follow-up outcomes, offering valuable insights for clinicians, policymakers, and researchers.

## 6. Conclusions

In conclusion, CD is a major health problem that requires lifelong management and can lead to serious complications if left untreated. Various screening strategies have been implemented in order to prevent these complications. Despite the unquestionable benefits of diagnosing CD, there is still no consensus or guidelines supporting mass screening of the general population. Therefore, further studies are needed that evaluate the clinical utility, cost-effectiveness, most appropriate target age, frequency of screening, optimal screening method, and long-term outcomes of mass screening strategies, particularly in asymptomatic individuals.

## Figures and Tables

**Table 1:** Studies included in this review.

	Region	Years of Study	Studied Population	Type of Study	Screening Method	Seropositive	Prevalence of CD	Ref.
1	Rep. of San Marino	1993–2009	5092 schoolchildren aged 6, 10, and 14 years	Mass screening study	AGA IgA/IgG tTG IgA followed by EMA IgA	AGA 0.70% tTG 1.80%	0.82%	[17]
2	Italy	2017–2020	4438 schoolchildren aged 5–11 years	Multicenter nationwide cross-sectional study (SIGENP study)	HLA-DQ2 and DQ8 (rapid test) followed by serum tTG IgA and total serum IgA	42.2%: HLA test + Seropositive: 4.28%. Prevalence of CD (in HLA+ children): 2.98%	1.65%	[18]
3	Italy	2015–2016	4570 schoolchildren aged 5–11 years	Screening study	HLA-DQ2 and DQ8 (rapid test) followed by total serum IgA, tTG IgA or DGP IgG and EMA IgA	43%: HLA test + Seropositive: 5.62%. Prevalence of CD (in HLA+ children): 5.62%	1.58%	[19]
4	Jordan	2006	1985 schoolchildren aged 5.5–9.5 years	Collaborative study between the Norwegian University and the Jordan University	tTG IgA followed by EMA IgA	tTG IgA 1.5% confirmation by EMA IgA 0.80%	0.80% No biopsy reported	[20]
5	Brazil	2009	1213 schoolchildren aged 11–17 years	Cross-sectional study	tTG IgA	0.49%	0.49% No biopsy reported	[21]
6	Northern region of Cyprus	2015–2016	3792 schoolchildren aged 6–10 years	Screening study	Total IgA tTG IgA/IgG EMA IgA	1%	0.39%	[22]
7	Saudi Arabia	2012–2014	1141 schoolchildren aged 6–18 years	Screening study	tTG IgA/IgG	2.80%	0.87%	[23]
8	Sweden	2005–2006	7567 schoolchildren, median age 12 years	Multicenter screening study (ETICS study phase I)	tTG IgA/IgG total IgA followed by EMA IgA/IgG	2.60%	3%	[24]
9	Turkey	2016 (date of publication)	1003 schoolchildren aged 5–18 years	Screening study	tTG IgA and total IgA (rapid test)	Rapid test was positive in 0.20%	0.30%	[25]
10	Malta	2020–2022	19,721 schoolchildren aged 3–13 years	Italia—Malta Cooperation Project (ITAMA project)	tTG IgM, IgA and IgG (rapid test) followed by total serum IgA, tTG IgA and EMA test	Rapid test was positive in 0.68% A final diagnosis of CD was made, according to the ESPGHAN guidelines, in 0.53% children who underwent screening by rapid test	0.42%	[26]
11	Finland	1994	3654 schoolchildren aged 7–16 years	Screening study	tTG IgA/IgG and EMA IgA/IgG followed by HLA-DQ	1.49%	1.01%	[27]
12	Iran	2013 (date of publication)	1500 schoolchildren aged 6–12 years	Cross-sectional screening study	tTG IgA total IgA	2%	0.60%	[28]
13	Switzerland	1999–2000	1450 schoolchildren aged 11–18 years	Screening study	Total IgA tTG IgA EMA IgA	0.75%	0.55%	[29]
14	Italy	1993–1995	17,201 schoolchildren aged 6–15 years	Multicenter screening study	AGA IgA/IgG EMA	7.50% (first-level test)	0.54%	[30]
15	Turkey	2016–2017	1730 schoolchildren aged 9–17 years	Screening study	tTG IgA and total IgA (rapid test) followed by tTG IgA/IgG EMA IgA/IgG	0.46%	0.46%	[31]
16	Turkey	2006–2008	20,190 schoolchildren aged 6–17 years	Screening study	tTG IgA/IgG EMA IgA/IgG	2.42%	0.47%	[32]
17	Tunisia	2003–2005	6284 schoolchildren	Screening study	tTG IgA and total IgA followed by EMA IgA	2.22% (initial screening test)	0.63%	[33]
18	Colombia	2015–2017	1402 schoolchildren aged 4–18 years	Cross-sectional multicity study	tTG IgA and total IgA (rapid test) followed by HLA-DQ	2.41% (initial screening test)	0.60%	[34]
19	Italy	2007–2010	5733 schoolchildren aged 5–8 years	Screening study	Salivary samples were tested for tTG IgA (fluid-phase radioimmunoassay) followed by serum tTG and EMA	0.73%	1.20%	[35]
20	Libya	2011 (date of publication)	2920 schoolchildren	Screening study	tTG IgA (rapid test) followed by serum tTG IgA	Rapid test positive 1.70%	0.69%	[36]
21	India	2022 (date of publication)	575 school-aged children	Cross-sectional study	tTG IgA	1.04%	1.04%	[37]
22	Hungary	2005	2690 children, median age 6 years (general population)	Screening study	tTG IgA (rapid test) followed by serum tTG IgA and EMA IgA/IgG	Rapid test positive 1.05%	1.38%	[38]
23	Italy	2021 (date of publication)	3307 children aged 1–14 years	Prospective study	tTG IgA (rapid test) followed by serum tTG IgA/IgG and total IgA	Rapid test positive 1.028%	0.33% children detected by rapid test	[39]
24	Mediterranean area (Italy, Slovenia, Turkey)	2014 (date of publication)	Italy: 3559 children (1–14 years), Slovenia: 1480 children (14–23 years), Turkey: 771 children (1–18 years)	Pragmatic study	tTG IgA (rapid test) followed by serology	Rapid test positive Italy: 3.11%, Slovenia: 1.21%, Turkey: 0.25%	Italy: 0.53%, Slovenia: 0.47%, Turkey: 0.12%	[40]
25	United States	1996–2001	1281 children aged 2–18 years	Multicenter study	AGA IgA/IgG EMA IgA tTG IgA	0.31%	0.15%	[41]
26	Hungary	1999 (date of publication)	427 preschool children aged 3–6 years	Screening study	EMA IgA/IgG	1.17%	1.17%	[42]
27	India	2003–2004	4347 schoolchildren aged 3–17 years	Screening study	tTG IgA	0.48%	0.32%	[43]
28	Germany	2003–2006	12741 children aged 1–17 years	Screening study (KIGGS study)	tTG IgA/IgG total IgA	0.80%	0.90%	[9]
29	Turkey	2008 (date of publication)	1000 children aged 2–18 years	Prospective study	tTG IgA and AGA (rapid test) followed by serum tTG IgA and EMA IgA	1%	0.90%	[44]
30	Turkey	2005 (date of publication)	1263 schoolchildren aged 6–17 years	Screening study	tTG IgA	0.87%	0.63%	[45]
31	Greece	2009	1080 preschool children aged 2–6 years	Multicity, multicenter study	tTG IgA and total IgA (rapid test) followed by serum tTG IgA and EMA IgA	0.65%	0.65%	[46]
32	Italy	2007	4048 schoolchildren aged 6–8 years	Screening study	Salivary samples were tested for tTG IgA (fluid-phase radioimmunoprecipitation method) followed by serum tTG IgA and EMA IgA	0.79%	1.16%	[47]
33	Croatia	2018–2019	1478 schoolchildren, median age 6 years	Screening study	tTG IgA (rapid test) followed by serum tTG IgA and total IgA	No child with a positive rapid test	No confirmed cases of CD	[48]
34	The Netherlands	2010–2013	4593 children, median age 6 years	Prospective study	tTG IgA followed by EMA IgA HLA DQ 2,2/2,5/8	1.35%	0.69%	[49]
35	Italy	1999–2000	3188 schoolchildren aged 6–12 years	Cross-sectional study	tTG IgA EMA IgA HLA DQ	1.50%	1%	[50]
36	Saudi Arabia	2014–2016	7930 schoolchildren aged 6–15 years	Prospective study	tTG IgA followed by EMA IgA	2.80%	1.50%	[51]
37	United Kingdom	2004 (date of publication)	5470 children, median age 7.5 years	Prospective study (ALSPAC study)	tTG IgA followed by EMA IgA	1%	1% (based on IgA-EMA test). Confirmatory biopsy was not possible	[52]
38	Vietnam	2015	1961 children aged 2–18 years	Screening study	tTG IgA followed by EMA IgA HLA DQ	1%	0.036% Confirmatory biopsy was not possible	[53]
39	United States	2017–2018	9973 children aged 1–17 years	Screening study (ASK study)	tTG IgA by two methods: radiobinding and electrochemiluminescence assay	2.42%	1.50%	[54]
40	India	2004	400 children aged 6 months–12 years	Screening study	tTG IgA	1.25%	1%	[55]
41	Argentina	2008–2009	2219 children aged 3–16 years	Cross-sectional study	tTG IgA followed by EMA IgA	1.30%	1.26%	[56]
42	Egypt	2001–2004	1500 children aged 7 months–18 years	Screening study	tTG IgA followed by EMA IgA total IgA tTG IgG	0.93%	0.53%	[57]
43	Estonia	1998–1999	1160 schoolchildren aged 9–15 years	Cross-sectional study	tTG IgA	0.43%	0.34%	[58]
44	Iran	2006–2008	634 schoolchildren aged 12–17 years	Cross-sectional study	tTG IgA total IgA	0.50%	0.50%	[59]
45	Sweden	1995–1996	690 children aged 2.5 years	Screening study	AGA IgA EMA IgA	0.86% both antibodies positive, 1.01% only EMA positive	1.30%	[60]
46	Italy	1997–1998	2096 schoolchildren aged 11–15 years	Screening study	AGA IgA/IgG EMA IgA Total IgA	0.57% AGA 0.76% EMA	0.91%	[61]
47	The Netherlands	1997–1998	6127 children aged 2–4 years	Screening study	EMA IgA	1.22%	0.50%	[62]
48	Russia Finland	1997–2001	1988 schoolchildren from Russia with a mean age of 11.6 years. 3654 schoolchildren from Finland with a mean age of 11.7 years	Cohort study	tTG IgA HLA DQ	0.60% Russia 1.40% Finland	0.20% Russia 0.93% Finland	[63]
49	Spain	2000–2002	830 children (First visit: 613 children aged 1.5 years. Second visit: 484 children aged 2.5%)	Prospective study	tTG IgA	First visit: 0% Second visit: 1.85%	1.44% (biopsy-proven CD at the second visit)	[64]
50	Spain	2009–2012	198 children aged 2–4 years	Cross-sectional study	AGA IgA and tTG IgA/IgG (rapid test)	3%	3%	[65]
51	Sweden	2005–2006	7567 schoolchildren aged 12 years	Screening study	tTG IgA/IgG total IgA followed by EMA IgA/IgG	2.60%	3%	[66]
52	Tunisia	2009	2064 schoolchildren	Screening study	tTG IgA and total IgA (rapid test) followed by serum tTG IgA and EMA IgA	0.34%	0.24%	[67]
53	Sweden	2005–2006 2009–2010	13,279 (7567 and 5712) schoolchildren aged 12 years	2-phase cross-sectional screening study (ETICS study)	tTG IgA/IgG total IgA followed by EMA IgA/IgG	In the 1993 cohort: 2.60% In the 1997 cohort: 1.90%	In the 1993 cohort: 3% In the 1997 cohort: 2.20%	[68]
54	Belgium	2006	1159 children and adolescents aged 1–19 years	Screening study	tTG IgA total IgA followed by EMA IgA/IgG DGP IgG	0.87%	No biopsy reported. Prevalence data of CD are not available	[69]
55	Saudi Arabia	2007–2008	1167 schoolchildren aged 16–18 years	Screening study	EMA IgA/IgG	2.20%	No biopsy reported. Prevalence data of CD are not available	[70]

CD: Celiac disease, tTG: tissue tranglutaminase, HLA: human leukocyte antigen, EMA: endomysial antibodies, AGA: anti-gliadin antibodies, DGP: deaminated gliadin peptide.

**Table 2:** Diagnostic accuracy of screening tests used (according to the studies included in the review).

Diagnostic Accuracy of Tests According to the Studies Included in the Review			
	Sensitivity	Specificity	Ref.
Biocard	Ranged from 78.1% to 97.8%	Ranged from >93% to 100%	[31,34,38,40,48]
Biohit	>99%	98.90%	[26]
Operon	CD 1WB 16.6%/CD 2WB 100%	89%	[65]
Salivary test	94.50%	98.20%	[47]
RBA	91–93%	98–100%	[54]
tTG IgA (ELISA)	90–99%	94–100%	[5]

tTG: tissue tranglutaminase, RBA: radiobinding assay.

**Table 3:** The cost of the various tests used to diagnose CD (according to the studies included in the review).

Assay	Cost According to the Studies Included in the Review
Rapid test (Biocard)	12.86 euros (15 USD) [34]
Rapid test (Biocard)	10 euros (11.65 USD) [38]
tTG IgA (ELISA)	15 euros (17.46 USD) [17]
EMA IgA	25 euros (29.10 USD) [17]
AGA IgG plus IgA	11.40 euros (12.81 USD) [17]
HLA test	300 euros (349.30 USD) [17]
Endoscopy	200 euros (232.87 USD) [17]
Total IgA	5 euros (5.82 USD) [17]

CD: celiac disease, tTG: tissue tranglutaminase, HLA: human leukocyte antigen, EMA: endomysial antibodies, AGA: anti-gliadin antibodies.

**Table 4:** Studies in which the initial screening was based on a rapid test.

Region	Detected by Rapid Test	Positive Rapid Test (According to the Study)	Prevalence of CD (According to the Study)	Ref.
Italy	HLA DQ2 and DQ8	42.20%	1.65%	[18]
Italy	HLA DQ2 and DQ8	43%	1.58%	[19]
Turkey	tTG IgA and total IgA (Biocard)	0.20%	0.30%	[25]
Malta	tTG IgM, IgA and IgG (Biohit)	0.68%	0.42%	[26]
Libya	tTG IgA	1.70%	0.69%	[36]
Hungary	tTG IgA and total IgA (Biocard)	1.05%	1.38%	[38]
Italy	tTG IgA and total IgA (Biocard)	1.03%	0.33% children detected by rapid test	[39]
Italy	tTG IgA and total IgA (Biocard)	3.11%	0.53%	[40]
Slovenia	tTG IgAand total IgA (Biocard)	1.21%	0.47%	[40]
Turkey	tTG IgA and total IgA (Biocard)	0.25%	0.12%	[40]
Turkey	tTG IgA and AGA IgA	1%	0.90%	[44]
Greece	tTG IgA and total IgA	0.65%	0.65%	[46]
Croatia	tTG IgA and total IgA (Biocard)	0%	No confirmed cases of CD	[48]
Turkey	tTG IgA and total IgA (Biocard)	0.46%	0.46%	[31]
Colombia	tTG IgA and total IgA (Biocard)	2.41%	0.60%	[34]
Tunisia	tTG IgA and total IgA	0.34%	0.24%	[67]
Spain	tTG IgA/IgG and AGA IgA (Operon)	3%	3%	[65]

CD: celiac disease, tTG: tissue tranglutaminase, HLA: human leukocyte antigen, AGA: anti-gliadin antibodies.

**Table 5:** Advantages and disadvantages of using a rapid test according to the studies in the review.

**Advantages** **(According to the Studies Included in the Review)**
Simplicity
Ease of performing the test even by non-medical personnel outside the hospital
Speed and immediacy of results
Improving the quality of life of patients due to early diagnosis
It is recommended for screening strategies in countries with limited resources
Much cheaper than the HLA test and biopsy
It is recommended for screening in specific social groups, such as the rural population and schoolchildren
More acceptable to parents and children than testing that requires venous blood draw
Allows immediate detection of positive cases, shortening the time to biopsy and treatment, reducing morbidity and mortality
Ideal method for mass screening strategies of asymptomatic, apparently healthy individuals
The unreliability of results due to time spent transporting or storing samples in laboratories is eliminated
Ease of repeating the test in case of an accident during the procedure or due to a doubtful result
Higher compliance of asymptomatic children in participating in mass screening using a rapid test compared to using a conventional TGA-IgA serological test
Useful as a screening tool for CD, especially in isolated areas without access to hospitals and laboratory tests
A mass screening strategy that uses a rapid test to screen asymptomatic children is a more convenient and efficient option than a symptom-based case-finding strategy
Additional costs of doctor visits, transportation costs, and loss of salary for parents if they have to take their child for a venous blood sample at a laboratory are avoided
Additional costs of exporting and delivering results are avoided
In the case of conducting the rapid test in schools, the loss of school days for children or workdays for parents is avoided
Early diagnosis offers social benefits, such as improving patients’ quality of life and reducing the insurance burden
**Disadvantages** **(According to the Studies Included in the Review)**
False-positive and false-negative results are not excluded, and confirmation of the diagnosis should always be followed
Since the test is qualitative, there is a possibility of unintentional error
The cost of purchasing rapid testing equipment and consumables
When calculating the cost of rapid testing per patient, the additional cost from possible repeat procedures, in case of an accident during the procedure or due to a doubtful result, must be added
Different interpretation of faint test lines may lead to different PPVs
In order to minimize the costs caused by false-positive results, it is advisable for the procedure to be supervised by a pediatric gastroenterologist with experience in these tests

CD: celiac disease, tTG: tissue tranglutaminase, HLA: human leukocyte antigen, PPV: positive predictive value.

## Data Availability

The original contributions presented in this study are included in the article. Further inquiries can be directed to the corresponding authors.

## Supplementary Materials

The following supporting information can be downloaded at: https://www.mdpi.com/article/10.3390/medicina62020246/s1, Table S1. Characteristics of the Studies included in the Systematic Review; PRISMA checklist. Table S2: Follow-up (according to the studies included in the review).
