# Supplementary material for: Mass Screening Strategies for Celiac Disease in Apparently Healthy Children and Adolescents: A Systematic Review
Source: Medicina (Kaunas). 2026 Jan 24;62(2):246. doi: 10.3390/medicina62020246 (PMC12942272; doi:10.3390/medicina62020246)
Supplement: Supplementary file 1 [file medicina-62-00246-s001.zip › medicina-4101378-supplementary.pdf]

Table S1. Characteristics of the Studies included in the Systematic Review; PRISMA checklist.

Section and Topic	Item #	Checklist item	Location where item is reported
**TITLE**			Page
Title	1	Identify the report as a systematic review.	1
**ABSTRACT**			
Abstract	2	See the PRISMA 2020 for Abstracts checklist.	1-2
**INTRODUCTION**			
Rationale	3	Describe the rationale for the review in the context of existing knowledge.	2-3
Objectives	4	Provide an explicit statement of the objective(s) or question(s) the review addresses.	3
**METHODS**			
Eligibility criteria	5	Specify the inclusion and exclusion criteria for the review and how studies were grouped for the syntheses.	4-6
Information sources	6	Specify all databases, registers, websites, organisations, reference lists and other sources searched or consulted to identify studies. Specify the date when each source was last searched or consulted.	4,6
Search strategy	7	Present the full search strategies for all databases, registers and websites, including any filters and limits used.	4
Selection process	8	Specify the methods used to decide whether a study met the inclusion criteria of the review, including how many reviewers screened each record and each report retrieved, whether they worked independently, and if applicable, details of automation tools used in the process.	5-6
Data collection process	9	Specify the methods used to collect data from reports, including how many reviewers collected data from each report, whether they worked independently, any processes for obtaining or confirming data from study investigators, and if applicable, details of automation tools used in the process.	6
Data items	10a	List and define all outcomes for which data were sought. Specify whether all results that were compatible with each outcome domain in each study were sought (e.g. for all measures, time points, analyses), and if not, the methods used to decide which results to collect.	4-6
	10b	List and define all other variables for which data were sought (e.g. participant and intervention characteristics, funding sources). Describe any assumptions made about any missing or unclear information.	4-5
Study risk of bias assessment	11	Specify the methods used to assess risk of bias in the included studies, including details of the tool(s) used, how many reviewers assessed each study and whether they worked independently, and if applicable, details of automation tools used in the process.	N/A
Effect measures	12	Specify for each outcome the effect measure(s) (e.g. risk ratio, mean difference) used in the synthesis or presentation of results.	N/A
Synthesis methods	13a	Describe the processes used to decide which studies were eligible for each synthesis (e.g. tabulating the study intervention characteristics and comparing against the planned groups for each synthesis (item #5)).	6
	13b	Describe any methods required to prepare the data for presentation or synthesis, such as handling of missing summary statistics, or data conversions.	6
	13c	Describe any methods used to tabulate or visually display results of individual studies and syntheses.	6
	13d	Describe any methods used to synthesize results and provide a rationale for the choice(s). If meta-analysis was performed, describe the model(s), method(s) to identify the presence and extent of statistical heterogeneity, and software package(s) used.	6
	13e	Describe any methods used to explore possible causes of heterogeneity among study results (e.g. subgroup analysis, meta-regression).	N/A

Section and Topic	Item #	Checklist item	Location where item is reported
	13f	Describe any sensitivity analyses conducted to assess robustness of the synthesized results.	N/A
Reporting bias assessment	14	Describe any methods used to assess risk of bias due to missing results in a synthesis (arising from reporting biases).	N/A
Certainty assessment	15	Describe any methods used to assess certainty (or confidence) in the body of evidence for an outcome.	N/A
**RESULTS**			
Study selection	16a	Describe the results of the search and selection process, from the number of records identified in the search to the number of studies included in the review, ideally using a flow diagram.	6-32
	16b	Cite studies that might appear to meet the inclusion criteria, but which were excluded, and explain why they were excluded.	6-32
Study characteristics	17	Cite each included study and present its characteristics.	6-32 TABLE 1
Risk of bias in studies	18	Present assessments of risk of bias for each included study.	N/A
Results of individual studies	19	For all outcomes, present, for each study: (a) summary statistics for each group (where appropriate) and (b) an effect estimate and its precision (e.g. confidence/credible interval), ideally using structured tables or plots.	TABLES 1-4, FIGURES 1-3
Results of syntheses	20a	For each synthesis, briefly summarise the characteristics and risk of bias among contributing studies.	N/A
	20b	Present results of all statistical syntheses conducted. If meta-analysis was done, present for each the summary estimate and its precision (e.g. confidence/credible interval) and measures of statistical heterogeneity. If comparing groups, describe the direction of the effect.	N/A
	20c	Present results of all investigations of possible causes of heterogeneity among study results.	N/A
	20d	Present results of all sensitivity analyses conducted to assess the robustness of the synthesized results.	N/A
Reporting biases	21	Present assessments of risk of bias due to missing results (arising from reporting biases) for each synthesis assessed.	N/A
Certainty of evidence	22	Present assessments of certainty (or confidence) in the body of evidence for each outcome assessed.	N/A
**DISCUSSION**			
Discussion	23a	Provide a general interpretation of the results in the context of other evidence.	32-33
	23b	Discuss any limitations of the evidence included in the review.	39
	23c	Discuss any limitations of the review processes used.	39
	23d	Discuss implications of the results for practice, policy, and future research.	33, 34- 38, 40
**OTHER INFORMATION**			
Registration and	24a	Provide registration information for the review, including register name and registration number, or state that the review was not registered.	4

Section and Topic	Item #	Checklist item	Location where item is reported
protocol	24b	Indicate where the review protocol can be accessed, or state that a protocol was not prepared.	4
	24c	Describe and explain any amendments to information provided at registration or in the protocol.	4
Support	25	Describe sources of financial or non-financial support for the review, and the role of the funders or sponsors in the review.	40
Competing interests	26	Declare any competing interests of review authors.	40
Availability of data, code and other materials	27	Report which of the following are publicly available and where they can be found: template data collection forms; data extracted from included studies; data used for all analyses; analytic code; any other materials used in the review.	40

From: Page MJ, McKenzie JE, Bossuyt PM, Boutron I, Hoffmann TC, Mulrow CD, et al. The PRISMA 2020 statement: an updated guideline for reporting systematic reviews. BMJ 2021;372:n71. doi: 10.1136/bmj.n71.

This work is licensed under CC BY 4.0. To view a copy of this license, visit https://creativecommons.org/licenses/by/4.0/

Table S2. Follow-up (according to the studies included in the review).

Reference	Initial findings	Follow-up time	Findings / Outcomes
**Rf [18]**	69/1612 subjects had tTG IgA positivity	**6-month follow-up (on a gluten containing diet)**	6/69 refused second test. 4/69 had tTG IgA+ but EMA- at second test (isolated tTG IgA positivity). 11/69 had tTG IgA- and EMA- at second test (transient tTG IgA positivity).<br>48/69 had tTG IgA+ and EMA+ at both first and second testing.<br>(20/48 had tTG IgA>10 UNL and symptoms: CD. 28/48 had tTG IgA>10 UNL without symptoms or tTG IgA<10 UNL: Biopsy suggested: 23/28 Marsh lesions 2–3: CD; 1/28 Marsh lesions 0–1: Potential CD; 4/28 Refused biopsy).
**Rf [19]**	96/1706 subjects had tTG IgA positivity	**6-month follow-up (on a gluten containing diet)**	22/96 had tTG IgA+ but EMA- at second test (isolated tTG IgA positivity).<br>20/96 had tTG IgA- and EMA- at second test (transient tTG IgA positivity).<br>54/96 had tTG IgA+ and EMA+ at both first and second testing.<br>(13/54 had tTG IgA>10 UNL and symptoms: CD. 41/54 had tTG IgA>10 UNL without symptoms or tTG IgA<10 UNL: Biopsy suggested: 30/41 Marsh lesions 2–3: CD; 2/41 Marsh lesions 0–1: Potential CD; 9/41 Refused biopsy).
**Rf [27]**	46/3654 subjects had tTG IgA+ and EMA+, without symptoms	**At biopsy**	Biopsy suggested: 27/46 Marsh lesions 2–3: CD.
		**Second test at the time of biopsy**	24/27 had both EMA IgA+ and tTG IgA+.<br>1/27 had both EMA IgA- and tTG IgA-.<br><br>2/27 had EMA IgG+ and tTG IgG+ (IgA deficiency).
		**Follow-up testing (on a gluten containing diet)**	5 children who were initially positive, were negative on follow-up testing.
**Rf [29]**	Newly diagnosed CD cases	**3-month trial period (on a GFD)**	Subjective improvement in general condition was then reported. However, the diet was discontinued due to the high cost and difficulty in obtaining gluten-free products.
**Rf [35]**	—	**3, 6, 9, 12, 18, and 24 months (on a GFD)**	tTG IgA normalization after 6 months on a GFD.<br>5 subjects were withdrawn from follow-up. The remaining subjects strictly followed a GFD.
		**> 1 year of follow-up**	36 subjects increased their weight and height.<br>22%: Improvement in school performance.<br>26%: Reduction of episodes of upper respiratory infections.
		**12-month follow-up**	68% were salivary-test negative and 75% were ELISA tTG IgA negative.<br>No salivary or serological confirmation in 10 individuals with borderline salivary tTG IgA and in one individual with low tTG IgA titers.<br>No CD-related complications in individuals who followed a GFD.

**Rf [38]**	32 newly diagnosed CD cases	**6-month follow-up (on a GFD)**	Significant reduction in median serum EMA IgA (from 1:40 to 1:2,5).<br>Significant reduction in median serum tTG IgA (from 56,5 U/ml to 5,0 U/ml). Significant increase in mean haemoglobin values (from 120,7 g/l to 128,0 g/l).<br>Increase in body mass index values (from 14,7 to 15,3).<br>Parents reported better general health status of children compared to the period before the screening.
**Rf [43]**	Children with CD	**3-month follow-up and every 3 months thereafter**	All CD patients showed clinical improvement with a gluten-restricted diet.
**Rf [50]**	—	**6 and 9 years of age**	High variability in serum tTG IgA levels.<br>Serum tTG IgA levels in subclinical CD were not predictive for the severity of enteropathy.
		**3-year follow-up after screening**	61% of asymptomatic 6-year-old tTG IgA positive children developed subclinical CD.<br>0,2% of tTG IgA negative children developed CD within 3 years after the negative tTG IgA screening result at 6 years of age.
**Rf [51]**	Biopsy-proven CD cases	**9, 12, 18, and 24 months (on a GFD)**	Significant reduction in both IgA and IgG tTG within the first 12 months.<br>All biopsy-proven CD cases reported compliance with the GFD.
		**12–18 months of follow-up**	66,7% negative for anti-tTG.<br>33,3% still positive for anti-tTG but with much lower levels than before GFD.<br>Persistent low positivity in 28,5% after 18 months may be due to suboptimal compliance.
		**Clinical outcomes**	Symptom resolution in all 12 symptomatic children.<br>4 asymptomatic children positive for thyreoperoxidase antibodies at diagnosis tested negative after 1 year of GFD.
**Rf [55]**	242 tTG IgA positive children	**Follow-up (on a gluten-containing diet)**	108 confirmed RBA tTG positive and referred for follow-up.<br>37/108 biopsy-proven CD. 3/108 potential CD.<br>2/108 serologic CD (ESPGHAN criteria). 18/108 persistent autoimmunity.<br>2/108 tested negative at follow-up.<br>12/108 started a GFD empirically.<br>14/108 refused clinical follow-up.<br>Follow-up pending for 20/108 children.

CD: celiac disease, tTG: tissue tranglutaminase, HLA: human leukocyte antigen, EMA: endomysial antibodies, AGA: anti-gliadin antibodies, DGP: deaminated gliadin peptide, ESPGHAN: European Society of Paediatric Gastroenterology, Hepatology and Nutrition, GFD: gluten free diet, UNL: upper normal limit.
